# Increases in external cause mortality due to high and low temperatures: evidence from northeastern Europe

**DOI:** 10.1007/s00484-016-1270-4

**Published:** 2016-11-17

**Authors:** Hans Orru, Daniel Oudin Åström

**Affiliations:** 1 Department of Family Medicine and Public Health, University of Tartu, Tartu, Estonia; 2 Department of Public Health and Clinical Medicine, Division of Occupational and Environmental Medicine, Umeå University, Umeå, Sweden; 3 Centre for Primary Health Care Research, Department of Clinical Science, Lund University, Lund, Sweden

**Keywords:** Temperature-related mortality, External causes, Distributed lag non-linear models

## Abstract

The relationship between temperature and mortality is well established but has seldom been investigated in terms of external causes. In some Eastern European countries, external cause mortality is substantial. Deaths owing to external causes are the third largest cause of mortality in Estonia, after cardiovascular disease and cancer. Death rates owing to external causes may reflect behavioural changes among a population. The aim for the current study was to investigate if there is any association between temperature and external cause mortality, in Estonia. We collected daily information on deaths from external causes (ICD-10 diagnosis codes V00–Y99) and maximum temperatures over the period 1997–2013. The relationship between daily maximum temperature and mortality was investigated using Poisson regression, combined with a distributed lag non-linear model considering lag times of up to 10 days. We found significantly higher mortality owing to external causes on hot (the same and previous day) and cold days (with a lag of 1–3 days). The cumulative relative risks for heat (an increase in temperature from the 75th to 99th percentile) were 1.24 (95% confidence interval, 1.14–1.34) and for cold (a decrease from the 25th to 1st percentile) 1.19 (1.03–1.38). Deaths due to external causes might reflect changes in behaviour among a population during periods of extreme hot and cold temperatures and should therefore be investigated further, because such deaths have a severe impact on public health, especially in Eastern Europe where external mortality rates are high.

**Electronic supplementary material:**

The online version of this article (doi:10.1007/s00484-016-1270-4) contains supplementary material, which is available to authorized users.

## Background

The relationship between hot and cold temperatures and mortality is well established (Turner et al. 2012; Gasparrini et al. 2015). The effects of heat on health usually occur relatively shortly after temperatures start to increase, whereas the effects of exposure to cold may take longer to emerge (Anderson and Bell 2009). The temperature-mortality relationship has been investigated including (Rey et al. 2007; McMichael et al. 2008), as well as excluding, external causes (Anderson and Bell 2009; Guo et al. 2014; de’ Donato et al. 2015).

In Estonia, deaths due to external causes, such as traffic accidents, assault, fires, drowning, and other injuries, are the third largest cause of mortality, after diseases of the circulatory system and cancer (Lai et al. 2009). Death rates and unintentional injuries owing to external causes may reflect behavioural changes among a population and have been less investigated (Otte im Kampe et al. 2016).

Hajat et al. (2007) investigated the relationship between temperature and mortality due to external causes in the UK, and Basagana et al. (2011) investigated the relationship in Catalonia, Spain. Both studies reported high temperatures to be associated with increased mortality due to external causes. In addition, a recent review by Otte im Kampe et al. (2016) evaluated the relationship between summer temperatures and unintentional injuries. Increasing temperatures were associated with an increased risk of both unintentional and work-related injuries, in higher income countries.

Both heat and cold diminish our ability to carry out mental and physical tasks, and this may be the mechanism that leads to more injuries, as well as physiological stress responses (Makinen and Hassi 2009; Kjellstrom et al. 2016). Heat, as well as cold, stress alters our cognitive functions, e.g. decreases task planning accuracy, impairs executive function, and decreases reaction time (Taylor et al. 2015).

Given that external mortality is the third largest cause of death in Estonia, our aim with the current study was to investigate the relationship between temperature and external mortality.

## Data

Estonia is situated on the Baltic Sea and borders by land the Russian Federation (to the east) and Latvia (to the south) and by sea Finland (to the north). Estonia is in the northern part of the temperate climate zone, with warm, dry summers and fairly severe winters.

For the period 1997 to 2013, we collected daily temperature and mortality data. The Estonian Weather Service provided daily maximum temperature data, recorded at the Türi measuring station in central Estonia (Supplementary Fig. S1). We assumed this centrally located measuring station to represent temperature exposure throughout Estonia, as the relative risks (RR) from this station should be representative for Estonia as a whole (Oudin Åström et al. 2016). Mortality data among 1.4 million residents were acquired from the Estonian Causes of Death Registry database. For each death, we obtained age, gender, and date and cause of death. We only included ICD-10 diagnosis codes V00–Y99 (external causes) deaths in the analysis.

## Methods

To model the relationship between temperature and mortality, we assumed that the daily counts of deaths followed an overdispersed Poisson distribution and used the generalised additive model:

$$ \begin{array}{l}Yt\sim \mathrm{Poisson}\left(\mu t\right)\\ {} \log \left(\mu t\right)=\alpha +{\upbeta}_1Tt,\mathrm{l}+{\upbeta}_2{\mathrm{weekday}}_{\mathrm{t}}+{\upbeta}_3{\mathrm{holiday}}_{\mathrm{t}}+NS\left(\mathrm{trend},df=6\ \mathrm{per}\ \mathrm{year}\right),\end{array} $$

where Yt is the daily number of deaths from external causes, *α* the intercept, and β_1_Tt,l a vector of coefficients representing the non-linear, as well as a delayed relationship, between maximum temperature and mortality of the same day (Gasparrini 2014). A quadratic B-spline and a natural cubic spline were fitted for temperature and time lag, respectively. Regarding temperature, we used two equally spaced internal knots and for the time lag, two equally spaced knots on a log-scale. Weekday is a categorical variable for the day of the week and holiday a binary variable indicating public holidays. To take into account variability in mortality due to seasonality and longer term trends, we included a time trend (natural cubic spline), with six degrees of freedom per year (chosen by comparing Akaike Information Criteria for different degrees of freedom). We centred the analyses at 20 °C, as this was found to be the minimum mortality temperature for external causes.

R version 2.13.1 was used to create datasets of variables, and the package DLNM (Gasparrini 2011) for statistical modelling and the creation of graphical output.

## Results

Between 1997 and 2013, there were 28,964 deaths recorded resulting from external causes, which corresponded to 10% of the total number of deaths in Estonia (Table S1). Eighty-two percent of external mortality cases were below 65 years of age and 78% were male. These deaths were evenly distributed over the year and a decreasing trend over the study periods was observed (Figs. S2 and S3). Traffic accidents, injuries, assault, drownings, and fires caused the most fatalities. Descriptive statistics of the daily maximum temperature are presented in Table S2.

**Fig. 1:**
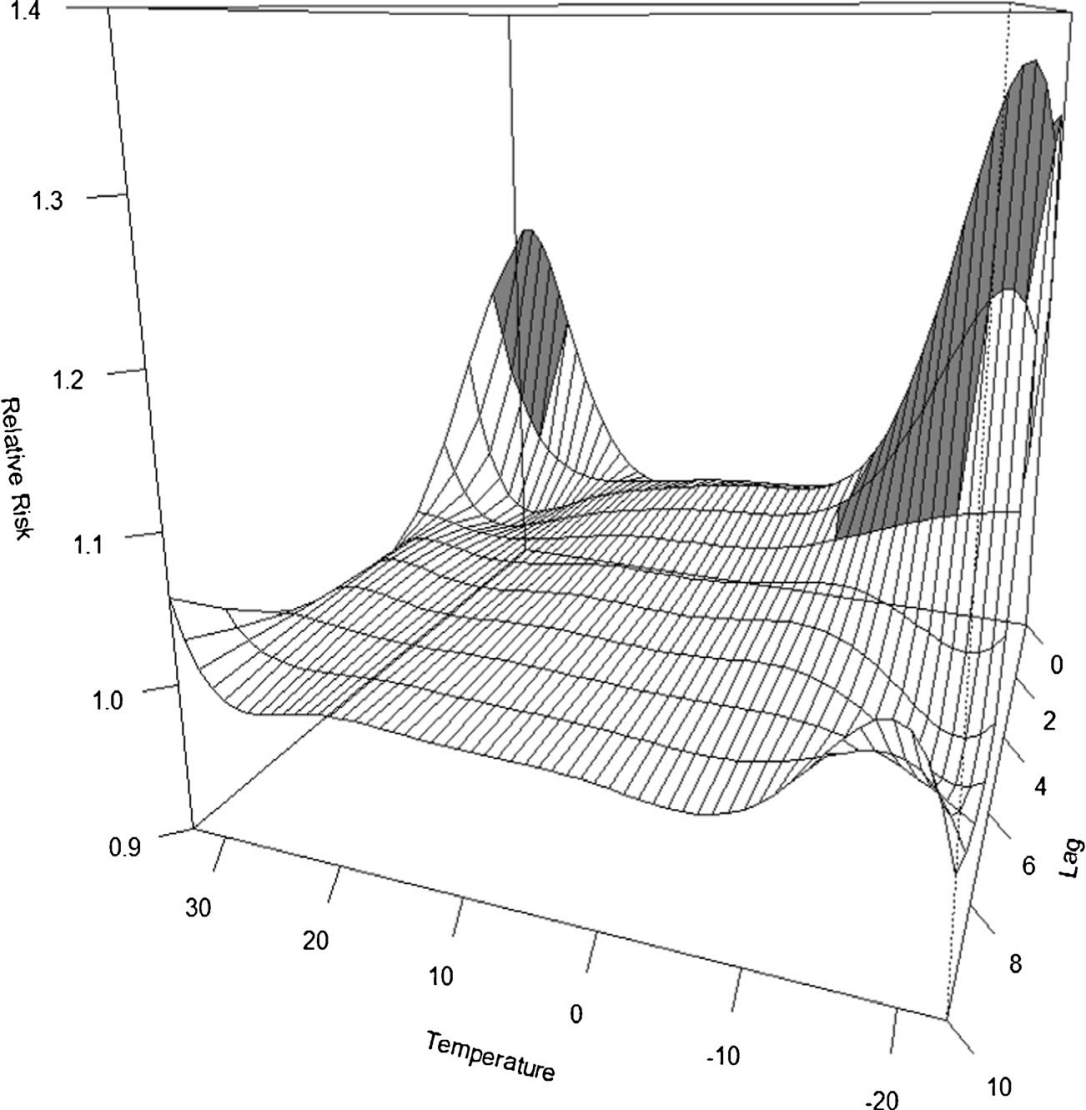
Relationship between external mortality and temperature in Estonia

We found significantly increased external cause mortality on hot (the same and previous day) and cold days (a lag of 1–3 days) (Fig. 1). The cumulative RRs for heat (an increase in temperature from the 75th/90th to 99th percentile (lag01)) were 1.24 (95% confidence interval, 1.14–1.35) and 1.18 (1.08–1.29), respectively. The cumulative RRs for cold (a decrease from the 25th/10th to 1st percentile (lag04)) were 1.18 (1.02–1.38) and 1.16 (0.99–1.36), respectively. The cumulative effects over lag04, are further illustrated in Fig. S4.

When stratifying the analyses, on age and gender, no statistically significant differences were observed; however, the point estimates for the under 18 population were of a larger magnitude. Traffic accidents increased for both, heat and cold, however, not statistically so, whereas the risk of death from assault (not significantly) and fires (significantly) seemed to increase during the cold season (Table S3).

## Discussion and conclusions

Any effects of extreme temperature on external cause mortality are not commonly expected to be delayed in time, and in our analysis, mortality due to external causes significantly increased shortly after exposure to high and low temperatures. Age and gender did not in our analyses modify the effect of high and low temperatures.

Our findings correspond with those of Hajat et al. (2007), who reported increased mortality due to external causes in England and Wales for 0–64 year olds (among whom most external mortalities occur) during hot weather. Our findings are also in-line with Basagana et al.’s (2011) report from the Catalonian region in Spain, where they found increased mortality due to external causes of a similar magnitude as we reported here.

Our study is among the first to show that extremely cold temperatures may increase mortality due to external causes, both totally and for fire-related mortality. It would be of interest if our finding of increased mortality due to assault during cold weather, even though it was not statistically significant due to the low number of deaths occurring in this category, could be replicated in other settings.

Traffic accidents accounted for the largest proportion of external mortality cases in Estonia, and we found increased risk due to extreme heat and cold, however, not statistically significant. Lately, Basagana et al. (2015) reported an increased risk of motor vehicle accidents during heat waves. Some recent studies support the inclusion of non-fatal injuries to quantify the total effect of heat exposure on health (Basagana 2014; Otte im Kampe et al. 2016).

As the majority of the deaths in the current study occurred among individuals of working age, one factor could also have been occupational accidents and injuries, as this has also been reported to be associated with high (Morabito et al. 2006; Xiang et al. 2014; Gubernot et al. 2015) and low temperatures (Morabito et al. 2014).

Deaths due to external causes might also reflect changes in behaviour among general populations, during periods of extreme temperature. Fralick et al. (2013) reported temperatures exceeding 30 °C to be associated with a 69% increase in the risk of outdoor drowning. High temperatures may increase the population at risk of drowning, as more people seek heat relief by outdoor bathing. In 2010 (the warmest summer ever in Estonia), there were 63 drownings in June and August, whereas in the following colder summers, only up to 34 drownings per annum were recorded (Estonian Rescue Board 2015).

In our analysis, we could not see any statistically significant results in individual categories, such as traffic accidents and assaults, owing to the low numbers of deaths occurring in each category (Table S3). Another limitation was that we assumed identical individual temperature exposure based on only one centrally located measuring station.

In areas with high external cause mortality rates, such as some Eastern European countries (Lai et al. 2009), excluding external cause of deaths, from the analyses, may lead to an underestimation of effects of temperature.

## Electronic supplementary material

Figure S1 (DOCX 550 kb)

Figure S2 (DOCX 60 kb)

Figure S3 (DOCX 60 kb)

Figure S4 (DOCX 57 kb)

Table S1 (DOCX 19 kb)

Table S2 (DOCX 18 kb)

Table S3 (DOCX 18 kb)

## References

[CR1] Anderson BG, Bell ML (2009). Weather-related mortality: how heat, cold, and heat waves affect mortality in the United States. Epidemiology (Cambridge, Mass).

[CR2] Basagana X (2014). High ambient temperatures and work-related injuries. Occup Environ Med.

[CR3] Basagana X, Escalera-Antezana JP, Dadvand P, Llatje Ò, Barrera-Gómez J, Cunillera J, Medina-Ramón M, Pérez K (2015). High ambient temperatures and risk of motor vehicle crashes in Catalonia, Spain (2000–2011): a time-series analysis. Environ Health Perspect.

[CR4] Basagana X, Sartini C, Barrera-Gómez J, Dadvand P, Cunillera J, Ostro B, Sunyer J, Medina-Ramón M (2011). Heat waves and cause-specific mortality at all ages. Epidemiology (Cambridge, Mass).

[CR5] de’ Donato FK, Leone M, Scortichini M, De Sario M, Katsouyanni K, Lanki T, Basagaña X, Ballester F, Åström C, Paldy A, Pascal M, Gasparrini A, Menne B, Michelozzi P (2015) Changes in the effect of heat on mortality in the last 20 years in nine European cities. Results from the PHASE project. Int J Environ Res Public Health 12(12):15567–15583. doi:10.3390/ijerph121215006 10.3390/ijerph121215006 PMC4690942 26670239

[CR6] Estonian Rescue Board (2015) Estonian Rescue Board yearbook 2014 Tallinn

[CR7] Fralick M, Denny CJ, Redelmeier DA (2013) Drowning and the influence of hot weather. PloS ONE 8(8) 10.1371/journal.pone.0071689 PMC3743751 23977112

[CR8] Gasparrini A (2011) Distributed lag linear and non-linear models in R: the package dlnm. J Stat Softw 43:1–20. doi:10.18637/jss.v043.i08 PMC3191524 22003319

[CR9] Gasparrini A (2014). Modeling exposure-lag-response associations with distributed lag non-linear models. Stat Med.

[CR10] Gasparrini A, Guo Y, Hashizume M, Lavigne E, Zanobetti A, Schwartz J, Tobias A, Tong S, Rocklöv J, Forsberg B (2015). Mortality risk attributable to high and low ambient temperature: a multicountry observational study. Lancet.

[CR11] Gubernot DM, Anderson GB, Hunting KL (2015). Characterizing occupational heat-related mortality in the United States, 2000–2010: an analysis using the census of fatal occupational injuries database. Am J Ind Med.

[CR12] Guo Y, Gasparrini A, Armstrong B, Li S, Tawatsupa B, Tobias A, Lavigne E, Coelho MSZS, Leone M, Pan X (2014). Global variation in the effects of ambient temperature on mortality: a systematic evaluation. Epidemiology.

[CR13] Hajat S, Kovats RS, Lachowycz K (2007). Heat-related and cold-related deaths in England and Wales: who is at risk?. Occup Environ Med.

[CR14] Kjellstrom T, Briggs D, Freyberg C, Lemke B, Otto M, Hyatt O (2016). Heat, human performance, and occupational health: a key issue for the assessment of global climate change impacts. Annu Rev Public Health.

[CR15] Lai T, Habicht J, Kiivet R-A (2009). Measuring burden of disease in Estonia to support public health policy. Eur J of Public Health.

[CR16] Makinen TM, Hassi J (2009). Health problems in cold work. Ind Health.

[CR17] McMichael AJ, Wilkinson P, Kovats RS, Pattenden S, Hajat S, Armstrong B, Vajanapoom N, Niciu EM, Mahomed H, Kingkeow C, Kosnik M, O’Neill MS, Romieu I, Ramirez-Aguilar M, Barreto ML, Gouveia N, Nikiforov B (2008). International study of temperature, heat and urban mortality: the ‘ISOTHURM’ project. Int J Epidemiol.

[CR18] Morabito M, Cecchi L, Crisci A, Modesti PA, Orlandini S (2006). Relationship between work-related accidents and hot weather conditions in Tuscany (Central Italy). Ind Health.

[CR19] Morabito M, Iannuccilli M, Crisci A, Capecchi V, Baldasseroni A, Orlandini S, Gensini GF (2014). Air temperature exposure and outdoor occupational injuries: a significant cold effect in Central Italy. Occup Environ Med.

[CR20] Otte im Kampe, E, Kovats S, Hajat S (2016) Impact of high ambient temperature on unintentional injuries in high-income countries: a narrative systematic literature review. BMJ Open 6(2). doi:10.1136/bmjopen-2015-010399 10.1136/bmjopen-2015-010399 PMC4762150 26868947

[CR21] Oudin Åström D, Åström C, Rekker K, Indermitte E, Orru H (2016). High summer temperatures and mortality in Estonia. PLoS One.

[CR22] Rey G, Jougla E, Fouillet A, Pavillon G, Bessemoulin P, Frayssinet P, Clavel J, Hemon D (2007). The impact of major heat waves on all-cause and cause-specific mortality in France from 1971 to 2003. Int Arch Occup Environ Health.

[CR23] Taylor L, Watkins SL, Marshall H, Dascombe BJ, Foster J (2015). The impact of different environmental conditions on cognitive function: a focused review. Front Physiol.

[CR24] Turner LR, Barnett AG, Connell D, Tong S (2012). Ambient temperature and cardiorespiratory morbidity: a systematic review and meta-analysis. Epidemiology (Cambridge, Mass).

[CR25] Xiang J, Bi P, Pisaniello D, Hansen A, Sullivan T (2014). Association between high temperature and work-related injuries in Adelaide, South Australia, 2001-2010. Occup Environ Med.

