# Supplementary figures and images for: Increases in external cause mortality due to high and low temperatures: evidence from northeastern Europe
Source: Int J Biometeorol. 2016 Nov 17;61(5):963–6. doi: 10.1007/s00484-016-1270-4 (PMC5411405; doi:10.1007/s00484-016-1270-4)

Supplementary Figure S1. Türi meteorological station in Estonia.


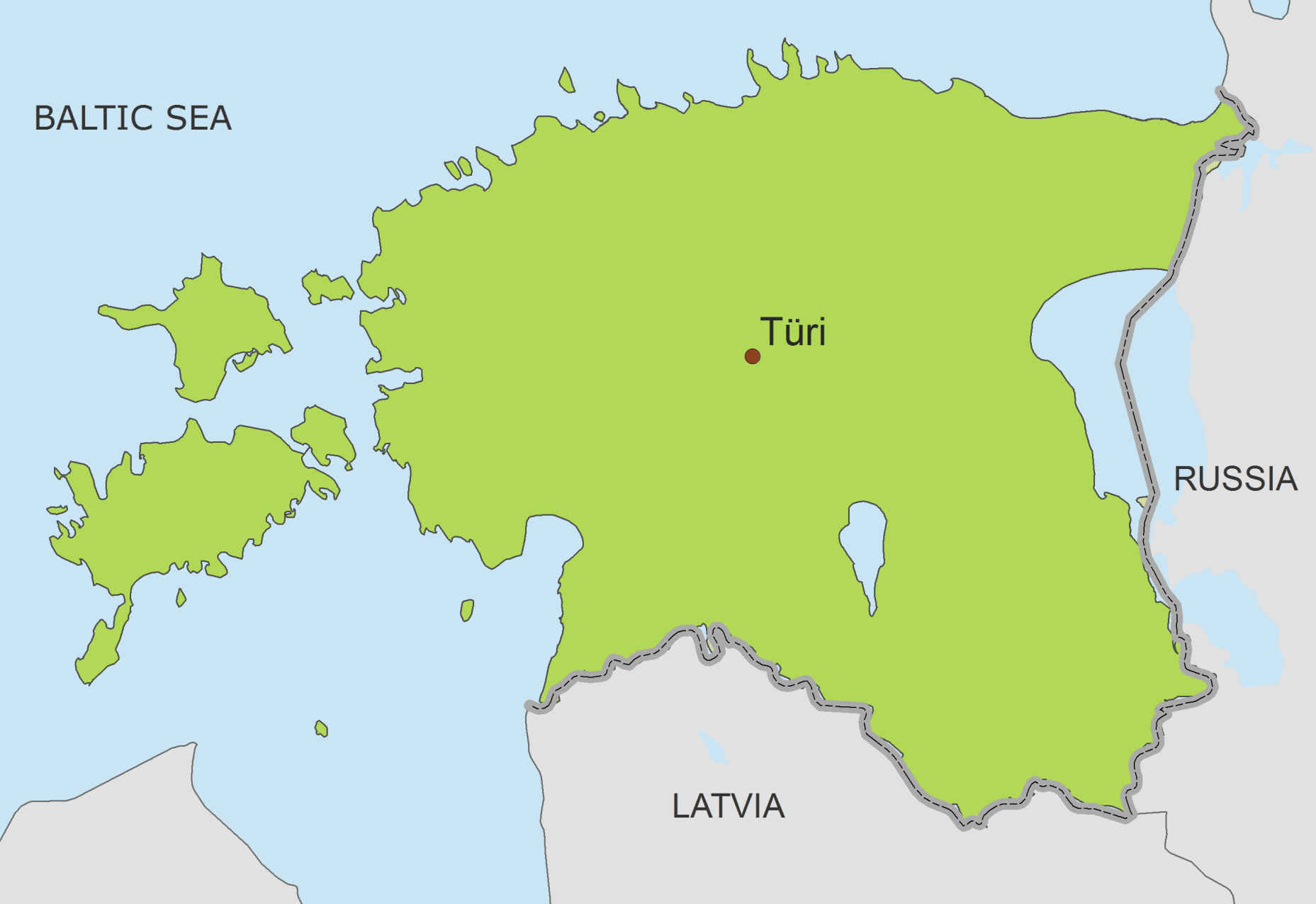

Supplement: Supplementary file 1 — (DOCX 550 kb) [file 484_2016_1270_MOESM1_ESM.docx]

Supplementary Figure S2. Number of deaths due to external causes presented by month.


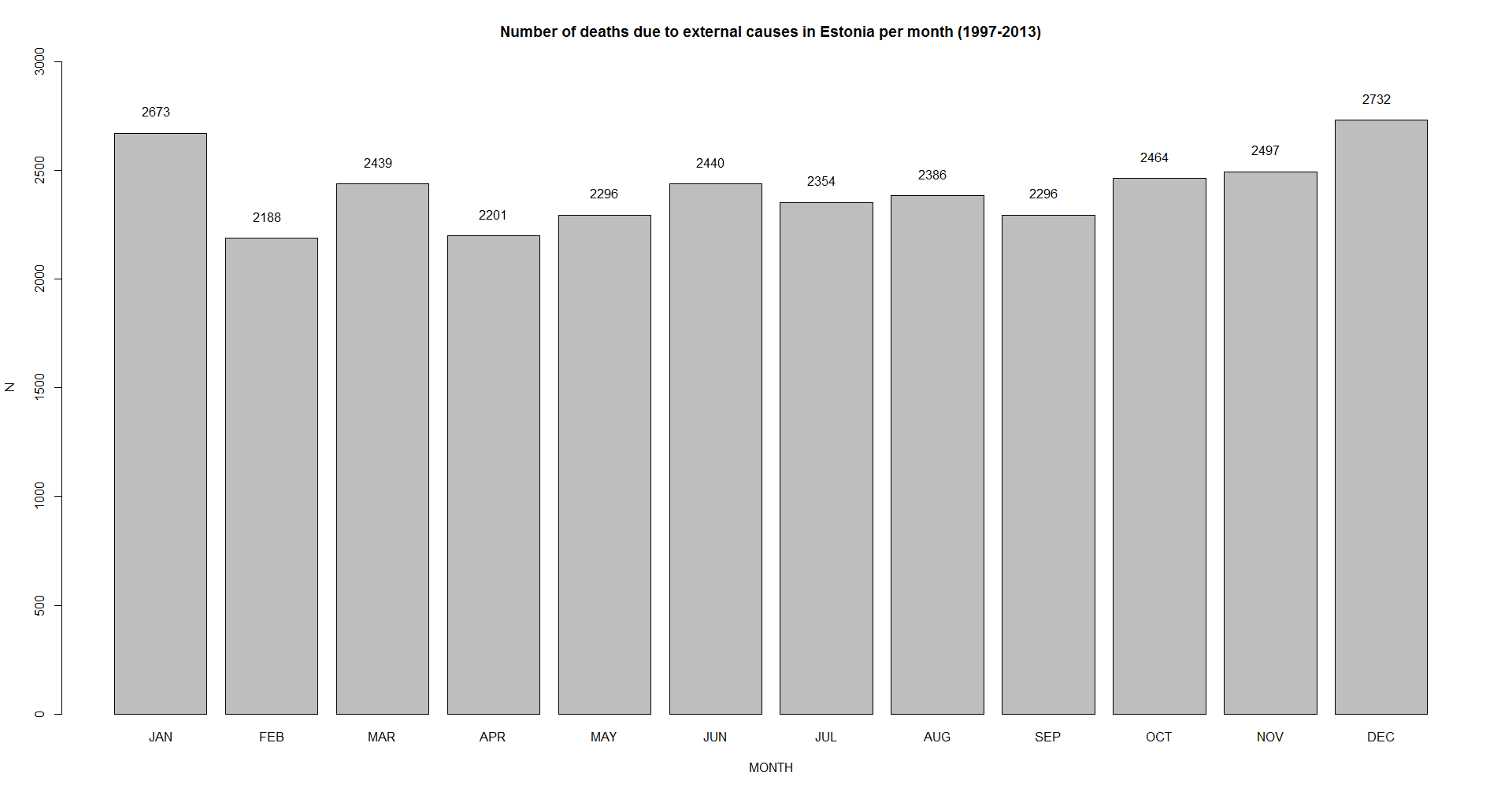

Supplement: Supplementary file 2 — (DOCX 60 kb) [file 484_2016_1270_MOESM2_ESM.docx]

Supplementary Figure S3. Number of deaths due to external causes presented by year.


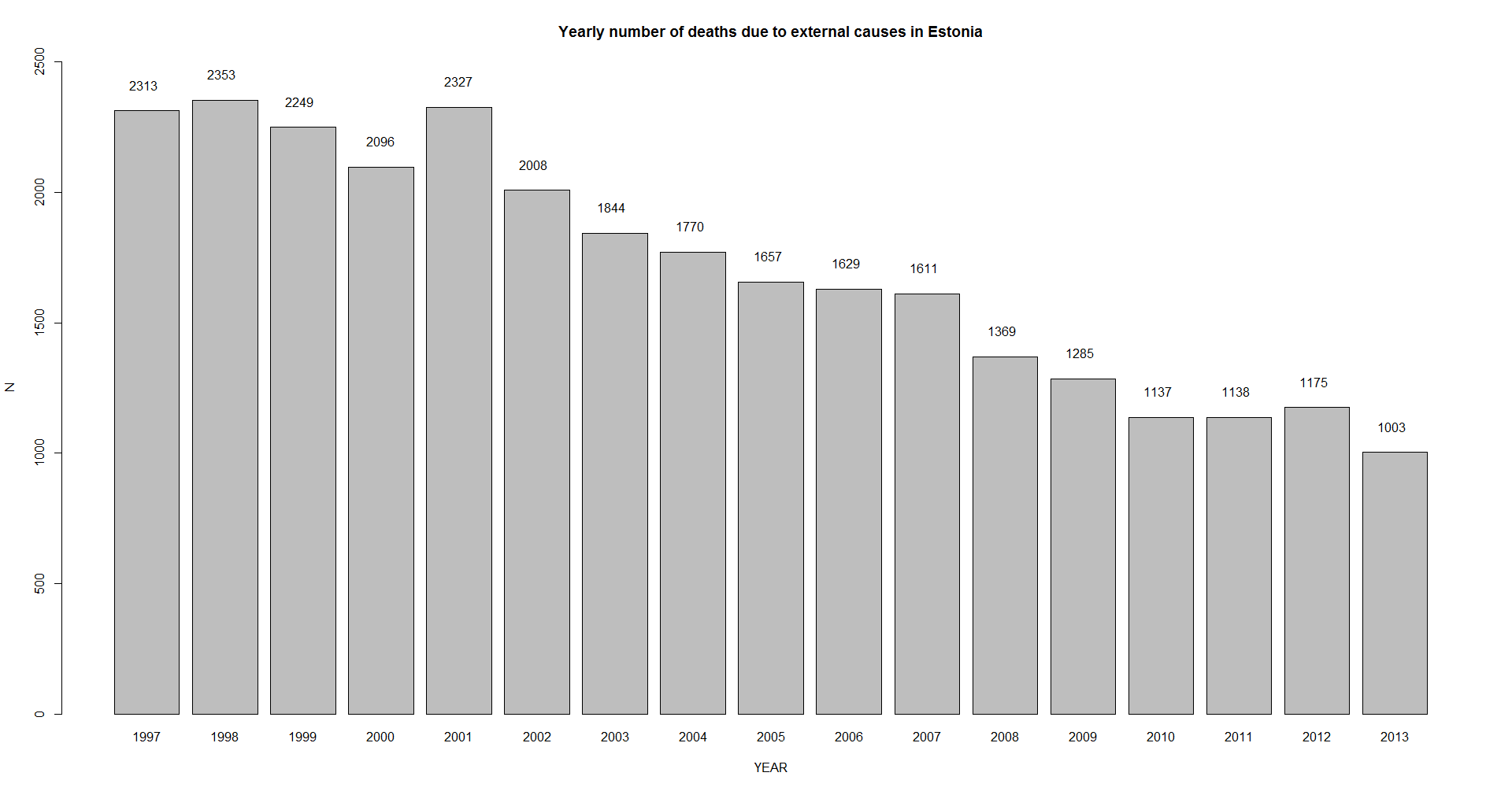

Supplement: Supplementary file 3 — (DOCX 60 kb) [file 484_2016_1270_MOESM3_ESM.docx]
