# Supplementary material for: Increases in external cause mortality due to high and low temperatures: evidence from northeastern Europe
Source: Int J Biometeorol. 2016 Nov 17;61(5):963–6. doi: 10.1007/s00484-016-1270-4 (PMC5411405; doi:10.1007/s00484-016-1270-4)

Supplementary Figure S4. The estimated cumulative RR of external mortality for daily maximum temperature, over lags 0 to 4


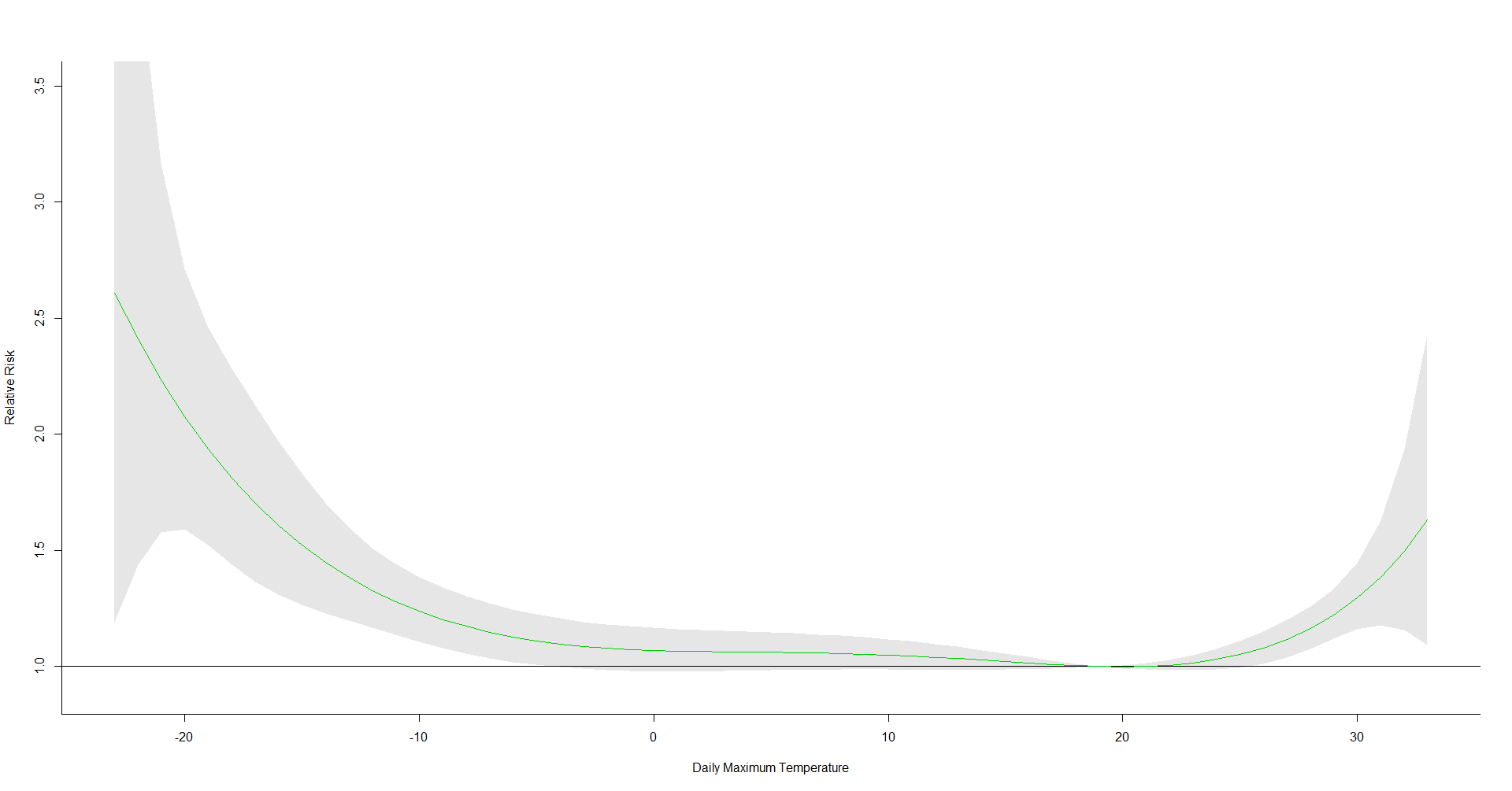

Supplement: Supplementary file 4 — (DOCX 57 kb) [file 484_2016_1270_MOESM4_ESM.docx]
