# Supplementary material for: Increases in external cause mortality due to high and low temperatures: evidence from northeastern Europe
Source: Int J Biometeorol. 2016 Nov 17;61(5):963–6. doi: 10.1007/s00484-016-1270-4 (PMC5411405; doi:10.1007/s00484-016-1270-4)
Supplement: Supplementary file 5 — (DOCX 19 kb) [file 484_2016_1270_MOESM5_ESM.docx]

Supplementary Table S1. Descriptive statistics of daily mortality due to external causes in Estonia 1997-2013

| Daily number of deaths (average of 1997–2013) | | | | | | Distribution of deaths due to external causes | | | | |
| --- | --- | --- | --- | --- | --- | --- | --- | --- | --- | --- |
| N Total | MEAN | SD | MEDIAN | MIN | MAX | % 0–17 | % 18-44 | % 45-64 | % 65 + | % Women |
| 28,964 | 4.8 | 2.6 | 4 | 0 | 20 | 5 | 36 | 40 | 19 | 22 |
| Daily number of deaths in the summer months,  May to September (average of 1997–2013) | | | | | | **Distribution of deaths due to external causes**  **during the summer** | | | | |
| N Total | MEAN | SD | MEDIAN | MIN | MAX | % 0–17 | % 18-44 | % 45-64 | % 65 + | % Women |
| 11,772 | 4.5 | 2.6 | 4 | 0 | 20 | 5 | 39 | 38 | 18 | 21 |
| Daily number of deaths in the winter months,  November to March (average of 1997–2013) | | | | | | **Distribution of deaths due to external causes**  **during the winter** | | | | |
| N Total | MEAN | SD | MEDIAN | MIN | MAX | % 0–17 | % 18-44 | % 45-64 | % 65 + | % Women |
| 12,527 | 4.9 | 2.7 | 5 | 0 | 20 | 5 | 34 | 42 | 19 | 24 |
| Daily number of deaths due to traffic accidents | | | | | | **Distribution of deaths due to traffic accidents** | | | | |
| N Total | MEAN | SD | MEDIAN | MIN | MAX | % 0–17 | % 18-44 | % 45-64 | % 65 + | % Women |
| 3452 | 0.6 | 0.9 | 0 | 0 | 10 | 8 | 49 | 28 | 15 | 23 |
| Daily number of deaths due to assault | | | | | | **Distribution of deaths due to assault** | | | | |
| N Total | MEAN | SD | MEDIAN | MIN | MAX | % 0–17 | % 18-44 | % 45-64 | % 65 + | % Women |
| 2281 | 0.4 | 0.7 | 0 | 0 | 6 | 6 | 20 | 45 | 29 | 26 |
| Daily number of deaths due to fire | | | | | | **Distribution of deaths due to fire** | | | | |
| N Total | MEAN | SD | MEDIAN | MIN | MAX | % 0–17 | % 18-44 | % 45-64 | % 65 + | % Women |
| 1913 | 0.3 | 0.7 | 0 | 0 | 10 | 4 | 46 | 38 | 12 | 23 |
| SD = standard deviation MIN = the minimum value observed during the period 1997–2013 MAX = the maximum value observed during the period 1997–2013  Traffic accidents = ICD-10 Codes V01-V99  Assault = ICD-10 Codes X85-Y09  Fire = ICD-10 Codes X00-X08 | | | | | | | | | | |
