# Supplementary material for: Increases in external cause mortality due to high and low temperatures: evidence from northeastern Europe
Source: Int J Biometeorol. 2016 Nov 17;61(5):963–6. doi: 10.1007/s00484-016-1270-4 (PMC5411405; doi:10.1007/s00484-016-1270-4)
Supplement: Supplementary file 6 — (DOCX 18 kb) [file 484_2016_1270_MOESM6_ESM.docx]

Supplementary Table S2. Descriptive statistics of daily maximum temperature data for Estonia 1997-2013

| Daily maximum temperatures (°C) during the whole year | | | | | | | | | |
| --- | --- | --- | --- | --- | --- | --- | --- | --- | --- |
| MEAN | SD | MIN | MAX | 1^st^ percentile | 10^th^ percentile | 25^th^ percentile | 75^th^ percentile | 90^th^ percentile | 99^th^ percentile |
| 10.2 | 10.3 | -23.0 | 33.4 | -12.0 | -2.8 | 1.8 | 19.1 | 23.5 | 29.2 |
| Daily maximum temperatures during the summer months | | | | | | | | | |
| MEAN | SD | MIN | MAX | 1^st^ percentile | 10^th^ percentile | 25^th^ percentile | 75^th^ percentile | 90^th^ percentile | 99^th^ percentile |
| 20.0 | 4.9 | 2.4 | 33.4 | 8.4 | 13.8 | 16.7 | 23.4 | 26.4 | 30.2 |
| Daily maximum temperatures during the winter months | | | | | | | | | |
| MEAN | SD | MIN | MAX | 1^st^ percentile | 10^th^ percentile | 25^th^ percentile | 75^th^ percentile | 90^th^ percentile | 99^th^ percentile |
| 0.3 | 5.4 | -23.0 | 17.4 | -15.0 | -6.5 | -2.7 | 3.7 | 6.7 | 11.2 |
| Measurements were based on one centrally located meteorological station, at Türi  Summer was defined as the months May to September  Winter was defined as the moths November to March  SD = standard deviation  MIN = the minimum value observed during the period 1997–2013  MAX = the maximum value observed during the period 1997–2013 | | | | | | | | | |
