# Supplementary material for: Increases in external cause mortality due to high and low temperatures: evidence from northeastern Europe
Source: Int J Biometeorol. 2016 Nov 17;61(5):963–6. doi: 10.1007/s00484-016-1270-4 (PMC5411405; doi:10.1007/s00484-016-1270-4)
Supplement: Supplementary file 7 — (DOCX 18 kb) [file 484_2016_1270_MOESM7_ESM.docx]

Supplementary Table S3. Cumulative Relative Risks with 95% Confidence Intervals for heat and cold

| Cumulative Relative Risks with 95% Confidence Intervals for heat (lag01) and cold (lag04) | | | | |
| --- | --- | --- | --- | --- |
|  | **HEAT EFFECT** | | **COLD EFFECT** |  |
|  | 75^th^ vs. 99^th^ | 90^th^ vs. 99^th^ | **25^th^ vs. 1^st^** | **10^th^ vs. 1^st^** |
| Total Mortality | 1.24 (1.14–1.34) | 1.18 (1.08–1.28) | 1.19 (1.03–1.38) | 1.17 (1.01–1.36) |
| 0-17 | 1.77 (0.98-3.20) | 1.43 (0.77-2.63) | 1.75 (1.14-2.68) | 1.71 (1.07-2.76) |
| 18-44 | 1.15 (0.92-1.44) | 1.25 (1.00-1.55) | 1.16 (0.97-1.40) | 1.13 (0.95-1.33) |
| 45-64 | 1.18 (0.94-1.47) | 1.17 (0.94-1.45) | 1.22 (1.05-1.42) | 1.14 (0.97-1.35) |
| 65+ | 1.17 (0.87-1.58) | 1.14 (0.84-1.54) | 1.17 (0.93-1.47) | 1.16 (0.94-1.42) |
| Male | 1.20 (1.03-1.40) | 1.18 (1.00-1.37) | 1.19 (1.06-1.33) | 1.17 (1.03-1.32) |
| Female | 1.27 (0.96-1.69) | 1.12 (0.84-1.50) | 1.22 (1.01-1.47) | 1.23 (1.00-1.52) |
| Traffic accidents | 1.44 (0.95-2.17) | 1.21 (0.79-1.85) | 1.05 (0.74-1.50) | 1.24 (0.84-1.83) |
| Assault | 1.02 (0.63-1.65) | 0.96 (0.59-1.58) | 1.13 (0.78-1.63) | 1.17 (0.78-1.77) |
| Fires | 0.97 (0.49-1.95) | 0.96 (0.47-1.97) | 1.49 (1.08-2.06) | 1.44 (0.99-2.09) |
